# Collecting wild potato species (*Solanum* sect. Petota) in Peru to enhance genetic representation and fill gaps in *ex situ* collections

**DOI:** 10.3389/fpls.2023.1044718

**Published:** 2023-01-30

**Authors:** Diego A. Sotomayor, David Ellis, Alberto Salas, Rene Gomez, Rosa A. Sanchez, Fredesvinda Carrillo, Carolina Giron, Violeta Quispe, Norma C. Manrique-Carpintero, Noelle L. Anglin, Cinthya Zorrilla

**Affiliations:** ^1^ Direccion de Recursos Geneticos y Biotecnologia, Instituto Nacional de Innovacion Agraria (INIA), Lima, Peru; ^2^ Facultad de Ciencias, Universidad Nacional Agraria La Molina (UNALM), Lima, Peru; ^3^ Centro Internacional de la Papa (CIP), Lima, Peru; ^4^ USDA ARS Small Grains and Potato Germplasm Unit, Aberdeen, ID, United States; ^5^ International Atomic Energy Agency, Plant Breeding and Genetics Section, Joint FAO/IAEA Center of Nuclear Techniques in Food and Agriculture, Vienna, Austria

**Keywords:** potato, crop wild relatives (CWRs), *ex situ* conservation, genetic resources, *Solanum* species, germplasm collecting

## Abstract

Crop wild relatives (CWRs) are important sources of novel genes, due to their high variability of response to biotic and abiotic stresses, which can be invaluable for crop genetic improvement programs. Recent studies have shown that CWRs are threatened by several factors, including changes in land-use and climate change. A large proportion of CWRs are underrepresented in genebanks, making it necessary to take action to ensure their long-term *ex situ* conservation. With this aim, 18 targeted collecting trips were conducted during 2017/2018 in the center of origin of potato (*Solanum tuberosum* L.), targeting 17 diverse ecological regions of Peru. This was the first comprehensive wild potato collection in Peru in at least 20 years and encompassed most of the unique habitats of potato CWRs in the country. A total of 322 wild potato accessions were collected as seed, tubers, and whole plants for *ex situ* storage and conservation. They belonged to 36 wild potato species including one accession of *S. ayacuchense* that was not conserved previously in any genebank. Most accessions required regeneration in the greenhouse prior to long-term conservation as seed. The collected accessions help reduce genetic gaps in *ex situ* conserved germplasm and will allow further research questions on potato genetic improvement and conservation strategies to be addressed. These potato CWRs are available by request for research, training, and breeding purposes under the terms of the International Treaty for Plant Genetic Resources for Food and Agriculture (ITPGRFA) from the Instituto Nacional de Innovacion Agraria (INIA) and the International Potato Center (CIP) in Lima-Peru.

## Introduction

Crop wild relatives (CWRs) are non-domesticated “wild” plant species that share a common ancestry with cultivated plants. They typically possess a wider range of genetic diversity in comparison with their cultivated counterparts due to their continued interaction with the environment and lack of genetic manipulation or selection by humans (Smýkal et al., 2018). CWRs offer a critical, often untapped resource, to address food security needs by providing genetic diversity of important agronomic traits for crop improvement, leading to increased plasticity and productivity of farming systems (Jansky et al., 2013). Genes found in CWRs can be introgressed into the crop by breeding programs (Harlan and de Wet, 1971; Singh, 2001; Castañeda-Álvarez et al., 2016) to provide traits such as pest and disease resistance, tolerance to abiotic stresses, increased yield, male fertility and quality, increasing the value and sustainability in crops (Dempewolf et al., 2017).

In the past 20 years, there has been a steady increase in the rate of cultivar releases containing genes from CWRs, and their contribution should only increase as the development of molecular technologies makes identification and utilization of diverse germplasm more efficient (Prescott-Allen and Prescott-Allen, 1986; Tanksley and McCouch, 1997; Singh, 2001; Hajjar & Hodgkin, 2007; Dempewolf et al., 2017). Plant breeders frequently obtain CWRs from genebanks. Having a representative diverse sample is of critical importance, especially at centers of origin for the crop, where diversity is the highest. However, major gaps in the genetic diversity of important crop gene pools such as potato (*Solanum tuberosum* L.), brinjal eggplant (*S. melongena* L.), and tomato (*S. lycopersicum* L.) remain to be filled in *ex situ* germplasm collections (Castañeda-Álvarez et al., 2015; Syfert et al., 2016; Vilchez et al., 2019). Moreover, the survival of some of these wild plant species are threatened by conversion of their natural habitats to agriculture, urbanization, invasive species, mining, climate change and/or pollution, and land-use change (Wilkes, 2007; Jarvis et al., 2008; Ford-Lloyd et al., 2011; Brummitt et al., 2015; Wettberg et al., 2022).

Potato (*S. tuberosum*) is the most important tuber crop worldwide, and one of the top staple foods in the world. According to FAOSTAT (2020) more than 20 million hectares globally are cultivated with potatoes, which produce more than 400 million tons. It continues to gain significance in temperate and tropical regions as a source of carbohydrates, vitamins, and minerals, as well as for industrial purposes (Navarre et al., 2009; Aversano et al., 2017). Potato wild relatives constitute a morphologically and genetically diverse group of plants and are geographically distributed from central Chile and Argentina to the southwestern United States covering 16 countries with high levels of endemism. Mexico, Bolivia, Argentina, and especially Peru, are considered to possess the greatest total diversity of potato wild relatives (Spooner and Salas, 2006) where they occupy a variety of habitats including deserts, forests, and mountainous regions (Hijmans et al., 2002). Potato is also one of the ten crops with the most breeding uses of CWRs documented (Dempewolf et al., 2017).

Currently, the taxonomic classification of tuber-bearing *Solanum* species (section Petota) recognizes 107 species of wild tuber-bearing potato species and four cultivated species (Spooner et al., 2014). From them, 53 species are distributed in Peru including 39 endemic species (Sarkinen et al., 2015). The taxonomy of potato is contentious as some conservation institutions still use older classification systems. For instance, the International Potato Center (CIP from its Spanish acronym) Genebank uses a classification based on the descriptions of Hawkes (1990) and Ochoa (1999) and has not yet adopted Spooner taxonomy (Spooner et al., 2014). Harmonization of the taxonomy used in global potato genebanks is needed for the extensive genetic comparison of potato collections to identify unique and redundant material between genebanks (Ellis et al., 2020).

The variety of ecosystems that are present in Peru, from north to south and from sea level to the rainforest, including seasonally humid habitats in the arid coast, locally known as “lomas”, have contributed to the evolution and survival of a vast variety of potato species. Wild potato species in Peru are diverse, not only in their geographical distribution, but also in their relative regional abundance and in the extensive gene flow that occurs between populations. Potato CWRs also vary widely in their life cycle duration, vegetative, and sexual reproduction (allogamous and autogamous species), time of flowering, pollination systems, as well as fruit and seed ripening (Salas et al., 2008; Ellis et al., 2020). Simulating these highly varied environmental conditions in *ex situ* conservation systems can be extremely challenging as many species may need specialized conditions (often unknown) to produce ample flowers, fruits, and seeds for genebank regeneration, conservation, and subsequent distributions of seed samples. Further, many species may not produce many flowers, fruits, and seeds even in their natural environments. Jansky et al. (2013) concluded that *ex situ* and *in situ* preservation are essential for a comprehensive conservation plan for potato CWRs and that collecting gaps detected in genebanks was a top priority. Castañeda-Álvarez et al. (2015), in an analysis of the state of *ex situ* conservation of potato CWRs, highlighted a high-priority need for the collection (*ex situ* gaps) of over 40% of the species of wild potatoes. This same study classified 26 species of wild potatoes native to Peru as high to medium priority for collecting. Thus, it is imperative that collection of these species is made before more habitats, populations, or species are lost.

To our knowledge there has not been any collections of wild potatoes (*Solanum* section Petota) for the past two decades or more in Peru, certainly not one that has placed the material into the Multilateral System (MLS) of the International Treaty for Plant Genetic Resources for Food and Agriculture (ITPGRFA) with materials readily available for the research community. Hence, there was and still is a dire need for plant collecting, as habitats are changing drastically throughout Peru due to urbanization, mining, and extremely rapid changes in the environment due to climate change, particularly affecting the Andes. This article presents the results of a series of wild potato collecting expeditions in Peru conducted under a global program led by the Global Crop Diversity Trust (GCDT) [for more specific details on the collecting in multiple crops and countries see (Eastwood et al., 2022)]. Here, we detail only the collection trips for potato in Peru, the biological material collected, the localities visited, the filling of *ex situ* conservation gaps, and discuss other specifics related to the collection and multiplication of the potato CWRs for long-term conservation in *ex situ* and routine distribution for research, training, and breeding.

## Materials and methods

### Study sites

**Figure 1:**
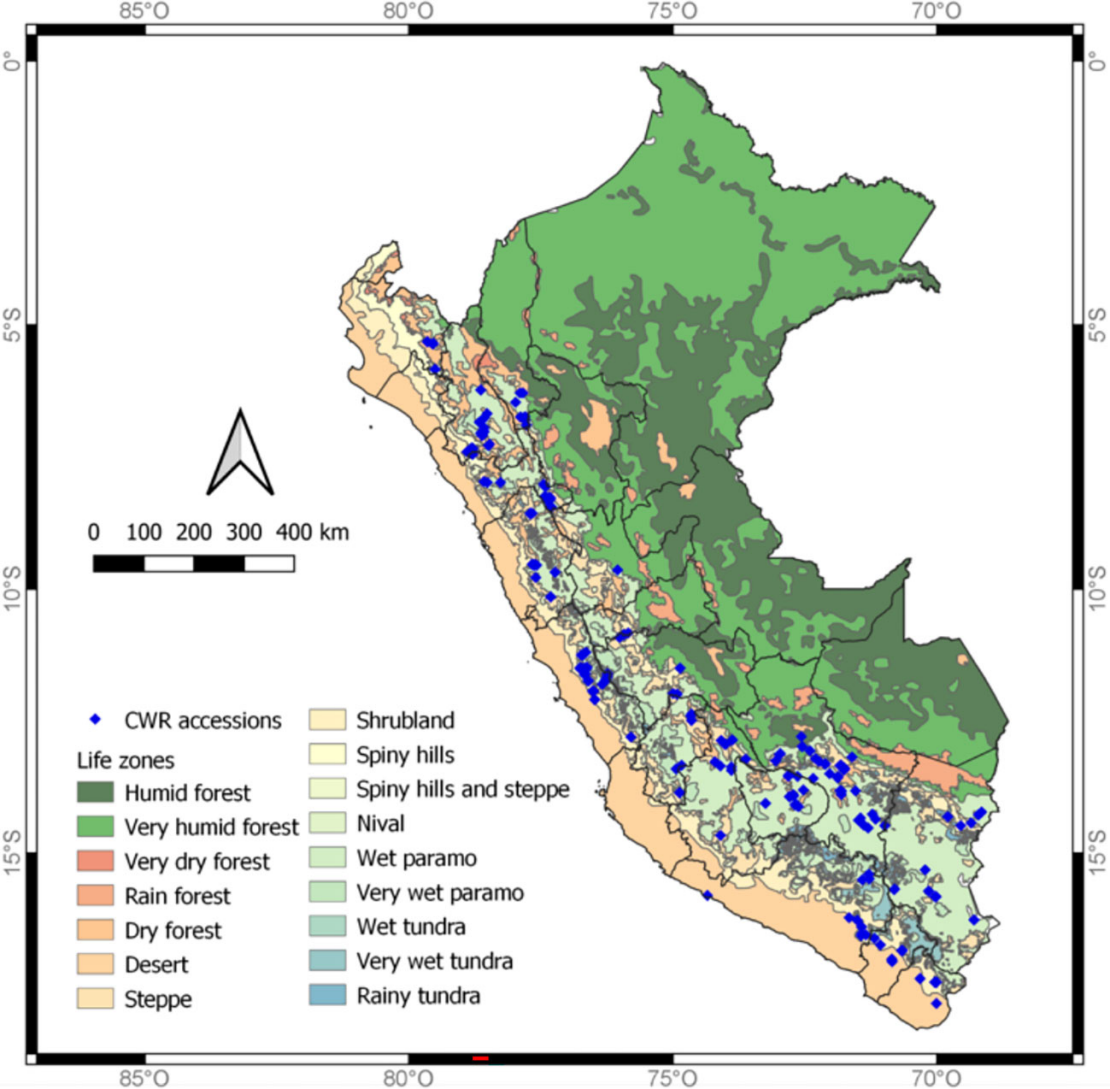
Map of potato CWRs accessions collected 2017/2018 in Peru with life zones (or habitats) depicted. The blue diamonds shown on the map are the sites in which the CWRs in this study were collected.

Potato CWRs are widely distributed in the Andean region of Peru (**Figure 1**); however, a few species occur in the arid coast, as well as in the Amazon basin. To determine the most appropriate timing and collecting route for each species, we based our decision on expert’s opinion and reported observation sites. This was complemented with the available data on the target species distribution as reported in global databases, such as Global Biodiversity Information Facility-GBIF (https://www.gbif.org/), Tropicos (https://www.tropicos.org/) and Genesys (https://www.genesys-pgr.org/), the CWR collecting guide (RBG Kew 2016), and the CIP Genebank database (https://genebank.cipotato.org/gringlobal/search.aspx). The former curator of wild potato species at CIP, Alberto Salas with ca. 50 years of experience collecting potato CWRs, led the planning of the collecting trips and provided detailed expertise on where potato species should be found based on previous collection trips in Peru. One goal was to re-visit sites where the species of interest had been collected decades prior to make new collections of the species, since genetic drift could be reshaping the allelic composition of many CWR species. The collection schedule was coordinated to target the best timing for as many populations as possible for most of the species and/or sites. Some of the collecting trips were performed in parallel with two crews going to separate geographic locations to maximize the potential of having a team be present during the growing season for collection. The crews of each trip were comprised by at least three researchers to help find the potato CWR plants and to record the data associated with the accessions collected.

### Germplasm collection

Prioritization of potato species for collecting included those species with low representation (from 0 to 10 accessions) in germplasm banks or herbariums globally. The main efforts for collecting were focused on three species, *S. ayacuchense*, *S. olmosense*, and *S. salasianum*. These species are endemic to Peru and do not exist in any genebank as reported by Castañeda-Álvarez et al. (2015), making them critical for collecting and *ex situ* conservation. In addition, species that were not represented or represented only by a single accession in the CIP Genebank were also priority targets for the collection trips. These include *S. arahuayum* (0 accessions), *S. jaenense* (0 accessions) and *S. ortegae* (1 accession). The taxonomic classifications developed by both Spooner et al. (2014) and Hawkes (1990) were used as an aid for targeting sites to visit. To determine synonymous species, the Solanaceae Source database (http://solanaceaesource.org/) was used in addition to the list of *Solanum* species in Peru (Sarkinen et al., 2015). Collecting for this project was conducted following Peru’s legal requirements, and hence, the Servicio Nacional Forestal y de Fauna Silvestre (SERFOR) authorized the collecting expeditions under permit number AUT-IFL-2016-038.

The unit of germplasm collected, or the accession, was a population defined as a set of plants growing near each other that belong to the same species and were at the same collecting site. A standard was set to have collection sites at least 2 km in distance from an adjacent one. Collections were made from areas walking distance of roads or paths, due to security and accessibility reasons.

The targeted collecting sample was mature berries containing mature seed obtained from at least five to ten plants. However, when berries were not present, tubers and/or whole plants from at least five to ten plants were collected for later greenhouse regeneration of seed. In addition, herbarium samples were taken to conserve and document a voucher of each population collected using a portable plant press. Following the instructions of the collecting permit granted by Peru´s national authority (SERFOR), “Free and Prior Informed Consent (PIC)” was obtained prior to collecting from any farmer´s community or private properties. Farmers were frequently consulted to help identify areas of interest for collecting.

A minimum set of passport descriptors that included collecting site and date, collectors, GPS coordinates, a brief description of the site (including biophysical characteristics and surrounding flora), a quick visual assessment of potential diseases and pests, and other features of interest, were recorded. Further, photographs of the material being collected, both in their natural habitat, as well as close-up of collected materials were taken and recorded in the database/collecting notes. The collecting notebook included the following data: collecting ID, collector names, genus, species, native name, state of the sample (wild, weedy), type of sample taken (voucher, fruits, tubers, seedlings, etc.), reported uses, pathogens, associated flora, topography, vegetation, and soil type.

### Sample processing

Mature berries were collected in paper bags and seed was extracted, washed, dried, and stored at CIP at -20°C according to the standard procedures established at CIP which is aligned with the international standards for seed conservation (FAO, 2014). In cases where the number of seed collected was below the minimum needed for conservation (6000 seeds per accession), seed was regenerated in greenhouses (CWRs are never multiplicated in a field setting due to the concern about invasive escapes). In cases where berries were collected but not fully mature, these were allowed to ripen prior to seed extraction by leaving them in paper bags at greenhouse temperature (20°C – 25°C). After seeds were extracted, they were split between the two collaborating institutions (INIA and CIP) for long-term conservation at -20°C in heat-sealed aluminium pouches.

When berries were not available, either tubers or whole plants were collected. Tubers were collected in paper bags, washed, and disinfected after arrival to the laboratory, and stored at 4°CC until needed for regeneration. Whole plants with roots and soil attached were collected, wrapped in moistened newspaper, and placed in plastic bags during transport back to CIP in Lima, Peru where they were immediately planted. Tubers and plants, were planted in 20 cm diameter pots, using Promix 8 as the substrate, grown in a screenhouse, and fertilized with Jack’s Professional fertilizer (3g/l before and 5g/l after flowering) and allowed to grow until mature plants with berries developed.

Herbarium samples consisted of whole plants pressed in a portable plant press on site using newspaper sheets between samples for transport back to the laboratory. Ideally, when possible, three samples were taken, the first sample for INIA, the second for CIP, and the third to be deposited in the Herbarium of the Natural History Museum in Lima, Peru. In the laboratory, the procedure for processing the herbarium samples started by carefully pressing, rapidly drying at 50°C for about 48 hours and then permanently mounting whole plant samples on herbarium sheets along with barcoded labels to identify each specimen and collecting information in the database. The herbarium sheets were then stored in polyethylene bags in controlled temperature (19°C-21°C) herbarium cabinets with controlled relative humidity (45-50%), following CIP standards for herbarium specimen conservation (https://cipotato.org/genebankcip/process/herbarium/, Vargas et al., 2016) prior to placement in the permanent herbarium collection.

### Seed regeneration

Seed regeneration was carried out for all accessions collected as tubers and/or whole plants, as well as for accessions with a low number of seed (less than 6000 seeds). Six thousand seeds were selected to ensure both *ex situ* institutions (INIA and CIP) maintaining this material, would have ample material for distribution requests and conserving the material into perpetuity. These accessions were grown under greenhouse conditions in Lima (coastal site), Huancayo, and Cusco (higher elevation sites) depending on the origin of the accessions and understanding of the best environmental conditions for regeneration.

In the case of seed collections needing a seed increase, a minimum of 100 seeds were germinated. A set of 25-30 seedlings were transplanted in Jiffys-7 pots for 30 days, and then transferred to 20 cm pots using a Promix 8 substrate (Salas et al., 2008). Due to the ISO 17025:2017 accreditation at CIP which regulates workflows to ensure that only virus free germplasm is moved around the globe, each seedling was tested for the following viruses using standard laboratory testing (ELISA, PCR, etc.) and complemented with biological indexing on indicator plants: Arracacha Virus B Oca strain (AVB-O), Alfalfa Mosaic Virus (AMV), Andean Potato Latent Virus (APLV), Potato Yellowing Virus (PYV), Tobacco Mosaic Virus (TMV), Potato Virus T (PVT), and the quarantine viroid Potato Spindle Tuber Viroid (PSTVd), after which only virus-free plants were used for seed regeneration. Any plants that were positive for these viruses were destroyed to prevent dissemination of viruses infecting potato.

When plants were flowering, depending on their mode of reproduction, autogamous or allogamous, open pollination or a combination of sib-crosses and bulk crosses were made, respectively, to obtain seed for conservation. The regeneration of seed was performed between 2017 and 2021. The resulting seed was divided and shared between CIP and INIA for long term conservation.

## Results and discussion

### Collecting expeditions

Prioritization of species and collecting sites was done *via* expert judgment and through a review of past collection sites, herbarium specimens, genebank passport data, and gap analysis results (Royal Botanical Garden Kew, 2016). An important aspect for collections such as these, is the identification of priority sites for *in situ* conservation of CWRs. It was observed that the habitat of potato CWRs are vanishing at an alarming rate, primarily due to changes in land use in Peru. Indeed, we found populations that were lost due to urban/agricultural expansion including *S. limbaniense* Ochoa in Cullucachi, Sandia-Puno, *S. saxatilis* Ochoa in Kkakkapata, Sandia-Puno, *S. medians* Bitter in San Juan de Lurigancho-Lima, *S. wittmackii* Bitter in Chorrillos-Lima, *S. mochiquense* Ochoa in Cerro Chipuitur and Cerro Cabras-Trujillo, and *S. olmosense* Ochoa in Porcuya, Huancabamba-Piura. In addition, populations are also lost due to intensified food production such as the development of chicken farms which has specifically impacted *S. chancayense* Ochoa and *S. inmite* Dunal in Chancay, Huaral-Lima. The loss of these populations emphasizes the need for continued collections and *ex situ* conservation of these valuable genetic resources.

In this sense, systematic conservation planning (Margules and Pressey, 2000) could be used to identify sites at a local scale and implement specific conservation actions to complement efforts at the global scale (Vincent et al., 2019). Comprehensive efforts, combining *ex situ* and *in situ* strategies employing multiple institutions working together are needed to conserve the genetic diversity of potato CWRs, especially in Peru, where large species diversity and ecogeographical conditions exist, especially because there are significantly increasing rates of land use change occurring in Peru.

A total of 18 collecting trips were conducted across a large portion of the varying habitats where potato CWRs have been documented or thought to be present in Peru. Where possible, trips included local INIA staff or farmers that guided and actively participated in the collection. The collecting trips are presented chronologically (**Table 1**) and locations are shown on a map (**Figure 1**). The collecting trips were performed at different times of the year between May-October 2017 and January-October 2018 to sample different life cycle stages of potato CWRs. The optimum time to collect a population is when they are in the later stages of flowering (with flowers desired for species identification and the collection of proper herbarium specimens) and ripe fruit present and still attached to the plant. Unfortunately, increased climatic uncertainty and consequent changes in patterns of development of plants made this condition harder to find than expected and, in most cases, either tubers or whole plants were collected, or a second visit was needed to collect the samples in the right stage of development. Effects of climate change disturbing crops in the Andes has been reviewed by Perez et al. (2010) - these factors also influence the development of their crop wild relatives.

**Table 1:** Collecting trips of potato CWRs performed in Peru during 2017/2018.

Trips	Regions	Collecting days	Accessions collected
1	Tacna, Moquegua, Arequipa, Apurimac, Ayacucho, Cusco	12	33
2	Puno, Tacna, Arequipa, Moquegua	12	4
3	Apurimac, Cusco	12	34
4	Trips suspended due to robbery, targeted Apurimac	0	0
5	Puno	10	19
6	Ayacucho, Huancavelica, Junín, Pasco	10	24
7	Pasco, Lima	11	64
8	Piura, Amazonas, Cajamarca	11	13
9	La Libertad, Ancash	11	38
10	Cajamarca, Amazonas, Piura	11	36
11	Huánuco, Ancash	7	2
12	Lima	3	1
13	Huancavelica	2	2
14	San Martín	5	0
15	Arequipa, Tacna, Moquegua	6	1
16	La Libertad, Lima	6	0
17	Lima	1	0
18	Cusco, Apurimac	11	51
Total	17	141	322

Unforeseen events limited the capacity to collect accessions over the course of 2017/2018. The main challenge was limited access to the north and central parts of the country in 2017 due to the occurrence of a coastal El Niño event bringing strong rains, landslides, and extensive road closures which were often unpaved and not well maintained (Rodríguez-Morata et al., 2019). As well, a severe drought after the coastal El Niño event in 2017, reduced the amount of rain in the coastal regions (also known as “lomas”) limiting plant development. To prevent situations that might affect the safety of the collectors, most collecting trips were rescheduled to 2018. The 2018 trips accounted for 105 collecting days, which in addition to the 36 days of collections in 2017, gave a total of 141 collecting days for the entire study. Other challenges that occurred included a landslide occurring in front of collectors on a road near the buffer zone of the Manu National Park in Cusco and an armed robbery of a collecting crew on the first day of the 2018 collecting campaign in Apurimac (central Peru), which resulted in some re-scheduling and delays of collecting activities along with loss of supplies and equipment needed for collecting. These unforeseen events, although not unique to the collection of potato CWRs or other crops for that matter, demonstrate the multitude of challenges that typically occur when conducting field research and the need for great flexibility in planning and travel. Atypical climatic events highlight the need for flexibility and future collecting trips to be planned to allow visits to sites in multiple years to ensure success and the preservation of target species before they vanish.

The collecting permit granted by SERFOR and the funding available for the project only allowed potato CWRs to be sampled over a two-year period so extension to another year was not possible. This was an additional challenge in collecting potatoes (time limitations). The collecting trips did not always unearth the prioritized species, but often other species were found in their place. This could be because the target species were either relatively rare or that the weather conditions were just not suitable for their emergence in the landscape during these years which is a typical challenge faced by plant collectors. For example, after the El Niño event of 1998, the population of *S. augustii* Ochoa in Ancash (central Peru) greatly increased making it possible to collect (Alberto Salas, pers. Comm.). Another challenge when visiting sites explored 20-40 years ago, was the effect of new human settlements and expansion of economic activities such as mining which have drastically changed the natural landscape in Peru and beyond. This resulted in some species becoming locally extinct. Thus, expanding the geographical area and time allotted for collecting particular potato species was greatly needed instead of making collections only near roads for efficiency and safety reasons. It is important to note that for this study, collecting sites could only be visited once or at maximum twice, and therefore, it was impossible to confirm if species still existed at a site or not, due to the time limitations imposed at each stop. Clearly, future collecting trips and their associated permits need to be carefully planned, optimally for more than a two-year time span to ensure the collection of species that may only germinate under certain climatic conditions. This flexibility should be recognized and supported by funding agencies.

Copies of the successfully collected material are deposited at both, INIA and CIP genebanks and are available for breeding, research, and training under the terms of the Standard Material Transfer Agreement (SMTA) of the ITPGRFA. In this context, the material collected is now available for use under the MLS and is safely conserved long-term in two genebanks in Peru. The collected material can be requested by users from CIP at (https://genebank.cipotato.org/gringlobal/search.aspx) or from the Agricultural Innovation Division of INIA (https://www.inia.gob.pe/requisitos-acceso-rrgg/), establishing a new chapter in the study of potato CWR.

### Seed regeneration and conservation

The amount of material collected that needed regeneration was greater than anticipated at the beginning of the study, in terms of (1) the number of accessions that required extensive seed regeneration for long-term storage; (2) the geographic locations where it had to be regenerated based on its origin, (3) the necessity to identify or validate the taxonomy of the accessions, and (4) the highly regimented virus testing procedures for accessions in order to only store, maintain, and distribute disease-free material. A considerable amount of care is required to meet these objectives in the regeneration effort, to maximize conservation of the representative genetic variation in the collected populations especially when small numbers of seed/genetic material were available. Another important point of consideration for planning future collection trips is building in sufficient time and labor for the effort required for extensive regenerations.

A total of 245 accessions, representing 76% of the 322 accessions originally collected, required regeneration. Regeneration of the material was initiated in September 2017 and finished in June 2021 at INIA and CIP greenhouses in Lima (coastal-central Peru), Huancayo (Andean-central Peru) and Cusco (Andean-southern Peru), with most of the regenerations performed in Huancayo. The decision to regenerate at a certain location was based on the daylength and elevation of the species natural habitat.

### Geographical representation of the collection

**Figure 2:**
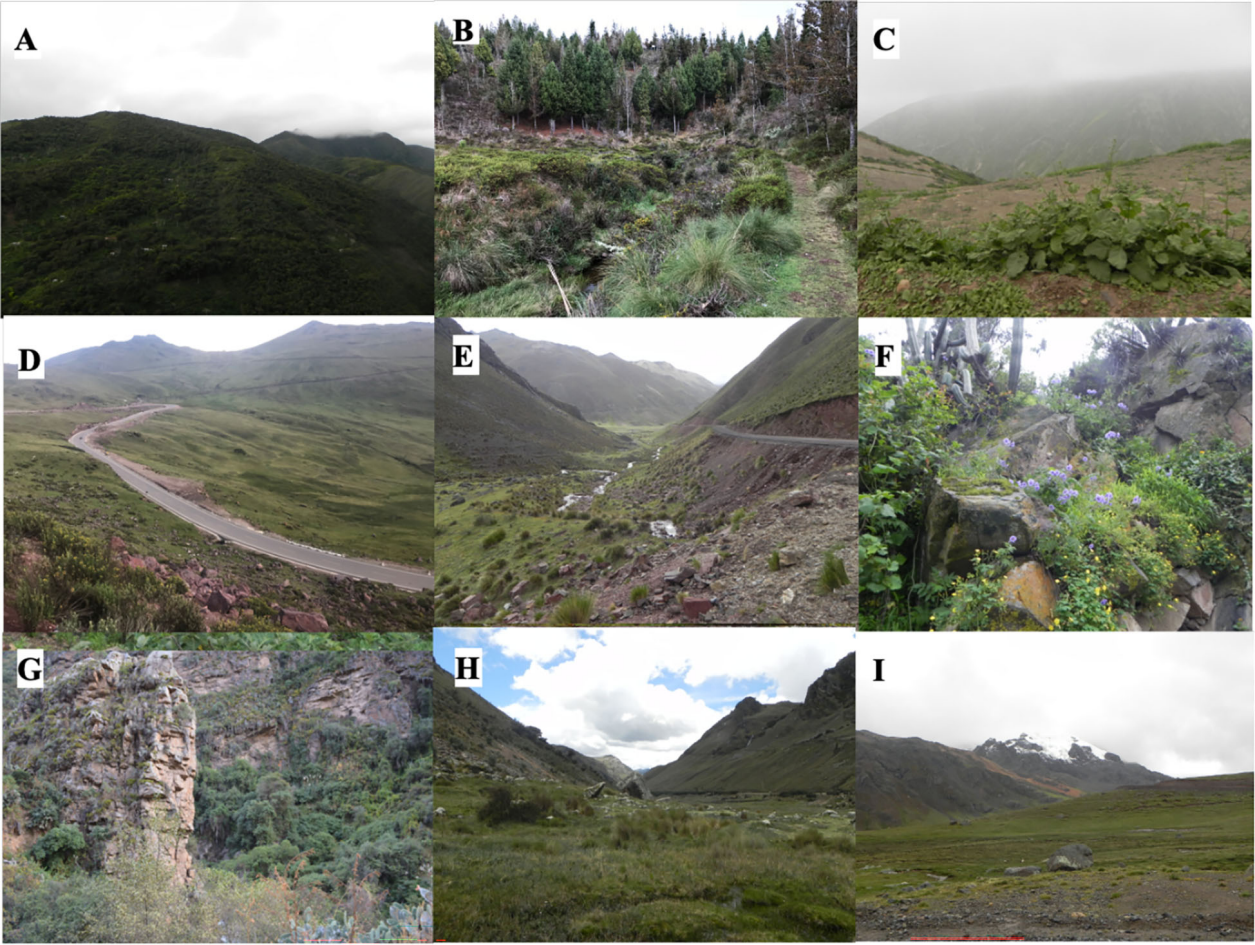
Habitats in Peru where potato CWRs were collected. **(A)** Humid and very humid forest habitats in Puno; **(B)** Dry forest habitat in Piura; **(C)** Desert habitats, lomas vegetation; Steppe habitats in **(D)** Ayacucho, and **(E)** Lima; **(F)** Shrubland habitats in Lima; **(G)** Spiny hills and steppe habitats in Apurimac; **(H)** Wet and very wet paramo habitats in Ancash; and **(I)** Tundra habitat in Pasco.

Our collecting trips acquired accessions from 17 out of the 25 political regions of Peru, regions not included were those located in the rainforest and the coastal desert, which are not known habitats of potato CWRs. The Peruvian Meteorological Service following Holdridge life zones (Holdridge, 1987) has divided Peru into 16 life zones: humid forest, very humid forest, very dry forest, rain forest, dry forest, desert, steppe, shrubland, spiny hills, spiny hills and steppe, nival, wet paramo, very wet paramo, wet tundra, very wet tundra, and rainy tundra. Collections were made in 12 (humid and very humid forests, dry forests, desert or arid environments, steppe, shrublands, spiny hills and steppe, very wet and paramo, and very wet and rainy tundra) of these specified zones (**Figures 1**, **2**). Wet paramo and steppe were the life zones from which more accessions were collected.

Castañeda-Álvarez et al. (2015) presented the regions and localities where high priority species were distributed, and all were included in the collecting campaign. However, as previously suggested, a more precise method for priorization of collecting sites and the identification of candidates for *in situ* conservation areas should be developed in the future to enhance the conservation efforts currently made by genebanks. Gap analysis (Ramirez-Villegas et al., 2010; Castañeda-Álvarez et al., 2015 at the global scale; Vilchez et al., 2019 at the national scale) and systematic conservation planning (Margules and Pressey, 2000; Vincent et al., 2019) that combine both herbarium/germplasm bank distribution data with species distribution modeling could be used for the priorization of sites for collecting and for *in situ* conservation purposes.

### Species representation in the collection

Taxonomic classification is a very important step for adequate conservation and utilization by researchers and breeders. A total of 322 accessions (populations) were collected between 2017 and 2018 of which 284 accessions were taxonomically classified belonging to 36 species according to Spooner et al. (2014), the respective species equivalency in Hawkes (1990) is also presented (**Table 2**). Additionally, 38 accessions have no species designation to date as certainty of the taxonomic identity of the species at the collection site or the greenhouse has not been possible and were thus labeled as *Solanum* spp. In this case, we propose that the complication with species identity could be due to the presence of natural hybrids. This highlights the need to further delineate species boundaries.

**Table 2:** The number of accessions collected and conserved, on a species basis, during eighteen collecting trips in Peru (2017/2018).

Spooner et al. (2014)	Hawkes (1990)	priority	collected	conserved
*S. acaule* Bitter	*S. acaule* Bitter	NFCR	35	33
*S. acroscopicum* Ochoa	*S. acroscopicum* Ochoa	HPS	10	9
	*S. lopez-camarenae* Ochoa	HPS	2	0
*S. albicans* Ochoa	*S. albicans* Ochoa	LPS	1	1
*S. amayanum* Ochoa	*S. amayanum* Ochoa	HPS	1	1
*S. ayacuchense* Ochoa	*S. ayacuchense* Ochoa	HPS	1	1
*S. boliviense* Ochoa	*S. megistacrolobum* Bitter	MPS	7	7
*S. brevicaule* Bitter	*S. sparsipilum* (Bitter) Juz. and Bukasov	MPS	8	7
*S. burkartii* Ochoa	*S. burkartii* Ochoa	HPS	5	2
*S. cajamarquense* Ochoa	*S. cajamarquense* Ochoa	HPS	7	7
*S. candolleanum* Berthault	*S. aymaraesense* Ochoa	HPS	2	2
	*S. bill-hookeri* Ochoa	HPS	1	1
	*S. bukasovii*	LPS	45	43
	*S. coelestispetalum* Vargas	MPS	3	3
	*S. longiusculus* Ochoa	HPS	2	1
	*S. marinasense* Vargas	MPS	7	4
	*S. orophilum* Correll	LPS	1	1
	*S. ortegae* Ochoa	HPS	1	1
	*S. pampasense* Hawkes	HPS	1	1
	*S. tarapatanum* Ochoa	HPS	1	1
	*S. velardei* Ochoa	HPS	2	0
*S. cantense* Ochoa	*S. cantense* Ochoa	HPS	2	2
*S. chacoense* Bitter	*S. yungasense* Hawkes	HPS	3	3
*S. chiquidenum* Ochoa	*S. chiquidenum* Ochoa	MPS	9	6
*S. chomatophilum* Bitter	*S. chomatophilum* Bitter	MPS	13	8
	*S. jalcae* Ochoa	HPS	4	2
*S. contumazaense* Ochoa	*S. contumazaense* Ochoa	HPS	1	1
*S. dolichocremastrum* Bitter	*S. dolichocremastrum* Bitter	MPS	3	3
*S. gracilifrons* Bitter	*S. gracilifrons* Bitter	HPS	1	1
*S. hastiforme* Correll	*S. hastiforme* Correll	HPS	1	0
*S. huancabambense* Ochoa	*S. huancabambense* Ochoa	MPS	1	1
*S. hypacrarthrum* Bitter	*S. guzmanguense* Whalen & Sagást.	HPS	1	1
*S. immite* Dunal	*S. immite* Dunal	HPS	3	1
*S. laxissimum* Bitter	*S. laxissimum* Bitter	HPS	5	3
	*S. santolallae* Vargas	HPS	1	0
*S. lignicaule* Vargas	*S. lignicaule* Vargas	HPS	3	3
*S. limbaniense* Ochoa	*S. limbaniense* Ochoa	HPS	2	1
*S. medians* Bitter	*S. medians* Bitter	MPS	17	15
	*S. sandemanii* Hawkes	HPS	7	6
	*S. tacnaense* Ochoa	HPS	12	8
*S. mochiquense* Ochoa	*S. mochiquense* Ochoa	HPS	2	2
*S. multiinterruptum* Bitter	*S. multiinterruptum* Bitter	LPS	14	14
*S. nubicola* Ochoa	*S. nubicola* Ochoa	HPS	2	2
*S. paucissectum* Ochoa	*S. paucissectum* Ochoa	LPS	4	0
*S. piurae* Bitter	*S. piurae* Bitter	HPS	3	3
*S. raphanifolium* Cárdenas & Hawkes	*S. raphanifolium* Cárdenas & Hawkes	LPS	12	12
*S. rhomboideilanceolatum* Ochoa	*S. rhomboideilanceolatum* Ochoa	HPS	2	2
*S. simplicissimum* Ochoa	*S. simplicissimum* Ochoa	HPS	1	1
*S. sogarandinum* Ochoa	*S. sogarandinum* Ochoa	MPS	1	1
*S. violaceimarmoratum* Bitter	*S. urubambae* Juz.	HPS	2	1
*S. wittmackii* Bitter	*S. wittmackii* Bitter	HPS	8	8
*Solanum* spp.	*Solanum* spp.	NA	39	34
36 species	51 species		322	238

Taxonomic identification as of August 2019. Collecting priorities (priority) according to Castañeda-Álvarez et al. (2015): high priority species (HPS), medium priority species (MPS), low priority species (LPS), no further collecting required (NFCR), not available (NA).

Collected species with reported traits of interest for breeding in cultivated potatoes include *S. acaule* Bitter, *S. boliviense* Dunal, *S. brevicaule* Bitter, *S. candolleanum* Berthault, *S. chacoense* Bitter, *S. chomatophilum* Bitter, *S. hypacrarthrum* Bitter, *S. medians*, *S. piurae* Bitter, *S. raphanifolium* Cárdenas & Hawkes, *S. sogarandinum* Ochoa and *S. violaceimarmoratum* Bitter. Traits include tolerance to below freezing temperatures, tolerance to semi-desert environments; resistance to viruses, pests and diseases such as late blight and others, and/or traits amenable to processing (Machida-Hirano, 2015; Kumari et al., 2018; Karki et al., 2021). The availability of a larger number of accessions for these species is an advantage for screening of the reported traits, as well as for the discovery of new traits of interest that may be mined for breeding purposes.

Future collecting efforts are still needed to fill genetic gaps and have a well-represented *ex situ* collection of potato CWRs conserved in genebanks. Although very successful, with this initial two-year collection effort, it was not possible to collect all high priority potato CWRs species. Examples of targeted species which were not found include *S olmosense* and *S. salasianum* Ochoa which are not represented in any genebank (Castañeda-Álvarez et al., 2015) and could be at risk due to their growth in specific habitats with a narrow geographical distribution. *S. olmosense* is distributed in the north of Peru (Lambayeque, Piura) and Ecuador (Loja) in the rain forests and *S. salasianum* is endemic to central Peru (Huanuco) in moist and humid habitats (Solanaceae Source, http://solanaceaesource.org/). Similarly, *S. arahuayum* Ochoa, *S. jaenense* Ochoa, and *S. ortegae* Ochoa (respectively considered synonyms of *S. medians*, *S. colombianum* Dunal and *S. candolleanum* by Spooner et al. (2014) were not found and are either not represented, or represented by a single accession, in the CIP Genebank.

It is important to note that the inability to find a species in this limited expedition cannot be inferred as evidence of species extinction as far more geographical surveillance is needed for such an assessment. Humphreys et al. (2019) reported two potato species as extinct, *S. cajamarquense* Ochoa and *S. hygrothermicum* Ochoa, both of which are native to Peru and this assessment had to be later corrected after evidence of their existence was demonstrated. *S. cajamarquense* is not extinct in the wild as it was collected during this collecting campaign, and in fact seed is available from multiple genebanks around the world (USDA, IPK, and CIP; see https://www.grin-global.org/, Genesys). In the case of *S. hygrothermicum*, Spooner’s revision of potato taxonomy did not recognize this species as valid, lumping it into *Solanum tuberosum* Andigena group; however, a specimen of this taxa was collected in 2006 and is available at the CIP genebank as an herbarium specimen. We agree, however with Humphreys et al. (2019), that extinction can be under or overestimated and that these caveats can be alleviated through increased study of poorly known biodiverse areas such as the Peruvian highlands.

Few exploratory plant collection trips have taken place in the relevant potato diversity areas since the entering into force of the Convention of Biological Diversity (CBD) and the bottleneck created by navigating the complex legal requirements to obtain the permission from a country through the required permits for collecting. Targeted searches of prioritized species need to be made in the future for multi-year collecting efforts to determine current distribution and conservation status, population dynamics and phenology, as well as for conservation of genetic resources in long-term *ex situ* storage.

### Filling the *ex situ* conservation gaps for potato CWR

**Figure 3:**
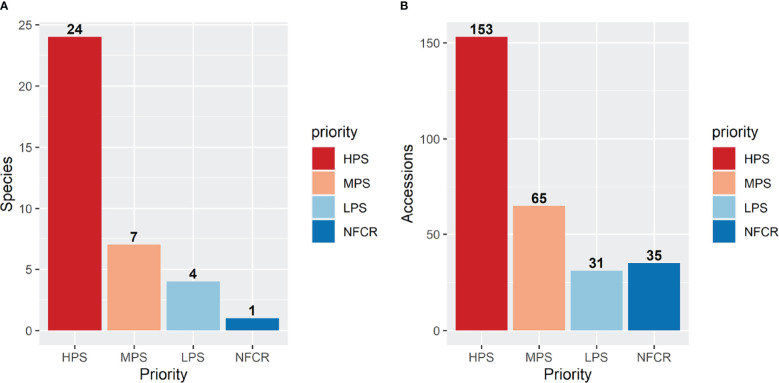
Number of species **(A)** and accessions **(B)** collected and conserved. Identification and prioritization of material for *ex situ* conservation was done using the Spooner et al. (2014) taxonomic classification. Collecting priorities according to Castañeda-Álvarez et al. (2015): high priority species (HPS), medium priority species (MPS), low priority species (LPS), and no-further collecting required (NFCR). Accessions with undetermined taxonomic classification (38 accessions) were not included in this figure.

**Figure 4:**
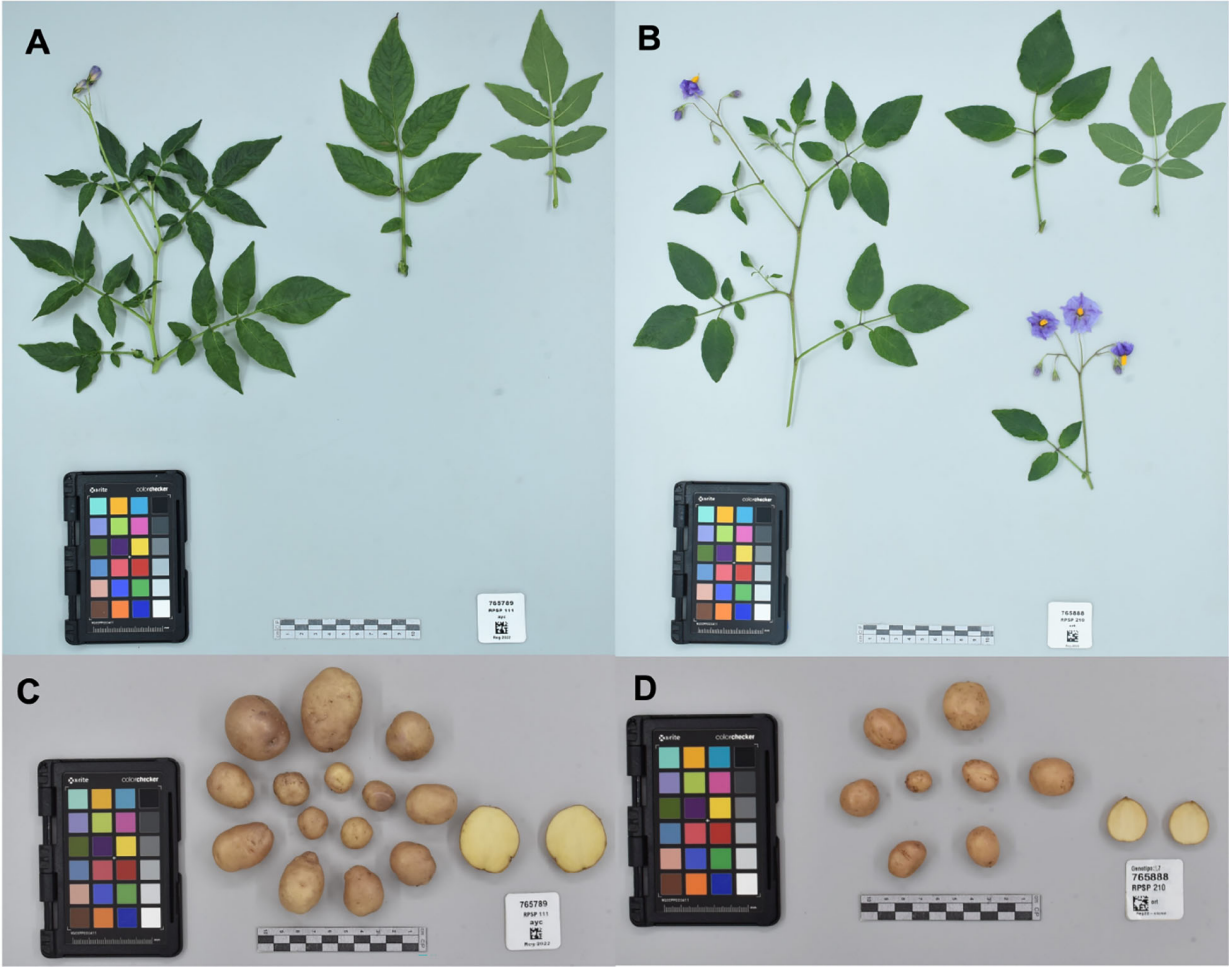
Herbarium specimen and tubers for: **(A, C)** *Solanum ayacuchense* Ochoa collected in Ayaerapampa, La Mar Province at the Department of Ayacucho. **(B, D)** *S. candolleanum* Berthault (*S. ortegae* Ochoa) collected in Paccaypata, Abancay Province at the Department of Huancavelica. No other accessions of these species are conserved in genebanks as far as the authors are aware. Photos taken by CIP’s Potato Genebank.

Accessions collected in this study will improve the representation of potato CWR conserved *ex situ* in Peru and globally, along with ensuring that they are publicly available under the terms of the SMTA from two genebanks, INIA and CIP; in addition, these accessions will be deposited at the Svalbard Global Seed Vault ensuring secure long-term conservation. However, there are several species endemic to other countries from the United States of America to Chile and Argentina that still need to be collected and conserved. Among the 36 species collected in this campaign for *ex situ* conservation, 24 species had high priority, seven had moderately high priority, four species with low priority, and one species (*S. acaule*) was not a priority for further collecting (**Table 2**). The *S. acaule* collections described here were mainly opportunistic (populations found while looking for another species). Clearly in this campaign, most accessions collected were high priority species indicating that the collecting efforts were successful in filling gaps of *ex situ* conservation (**Figure 3**). Of particular note is the gap filled by the collection of one *S. ayacuchense* Ochoa accession, a species with high conservation priority that had not been previously conserved in any genebank (**Figure 4**). Another important accession collected is a population of *S. candolleanum*, synonym of *S. ortegae* under Hawkes (1990) and Ochoa (1999) taxonomical treatment, also with high conservation priority and not available in any genebank until now (**Figure 4**).

### Recommendations for plant collection trips

Four major aspects that should be considered in future collecting campaigns include:

(1) Strong phenologic changes exist from year to year affecting the duration of growth cycles, the number of flowers formed, pollination efficiency and the number of berries formed. These changes would have a greater impact on CWRs than on their cultivated counterparts which could be further impacted by climate change and consequently will increase their unpredictability.
(2) Regeneration in greenhouses or field (if applicable) is necessary to conserve sufficient quantities of virus-free seed. Approximately 76% of the collected samples required further multiplication to ensure sufficient seed for conservation and distribution. Future collecting campaigns need to include several extra years after collection for multiplication and virus testing before material can be made available to researchers. Proper timing of collections to ensure the ability to collect ample samples at the time of collection and/or adequate regeneration success in the first regeneration cycle can avoid subsequent regenerations of small populations that could cause variation in the population structure and loss of key alleles.
(3) Local collaborators (farmers) are essential for successful collecting trips. Local inhabitants have unique knowledge of the geography and species habitats. Collaborations with local communities and/or farmers during collection improves the depth of searches, strengthens local capacities, and can significantly decrease the time required to organize collecting trips. This could also lead to an increased sense of the need of conservation of habitats locally. Further, local farmers tend to be invested in safeguarding plant material and understand the importance of conservation efforts for food security.
(4) Allowing sufficient time to navigate and obtain host country collecting permits and ensuring previous and informed consent (PIC) are obtained during collection process is of paramount importance to comply with obligations set forth from the Convention in Biological Diversity. The process of obtaining the permits can take quite a lot of time to navigate and may require building relationships and trust with key partners to ensure success of getting the necessary permits. The system for obtaining collecting permits can change country by country as it is not standardized, hence having host country partners can help point research to the necessary institutions and requirements for obtaining a permit. Funding agencies should also consider this aspect by giving flexibility to researchers.

## Conclusions

Between 2018 and 2019 we conducted 18 collecting trips accounting for 141 collecting days across the main distribution of potato’s CWR in Peru. This is the first comprehensive expedition in the last 20 years in a country that is center of origin of one of the most important crops in the world. We collected 322 CWR accessions belonging to 36 species of potato CWR, filling the gap for ex situ conservation (Castañeda-Álvarez et al., 2015) with 24 species designated as a high priority for collection, including *S. ayacuchense* that had not been previously conserved in any genebank until now. During the collection we found that the habitats of potato’s CWR are quickly disappearing, which underlines the importance of strengthening both *in situ* and ex situ conservation efforts.

The potato CWRs collected in this study have contributed to the reduction of genetic gaps in *ex situ* collections worldwide. Nevertheless, future collecting efforts are still needed to have a well-represented potato CWR *ex situ* collection conserved in global genebanks, preventing loss of valuable genetic materials due to changes in natural habitats. Further, more intensive collecting efforts could help document habitat loss and the possibility of associated extinctions of key species, highlighting the need to continually document changes and collect species before they are lost in their natural environments. Strategic conservation initiatives that combine *in situ* and *ex situ* conservation are necessary to ensure the sustainable conservation of potato CWRs into the future. This is further accentuated by the accelerated changes in the habitats of potato CWRs, as well as the effect of climate change, that could drive local extinction of some populations.

## Data availability statement

The datasets presented in this study can be found in online repositories. Data is available on GENESYS: https://www.genesys-pgr.org/subsets/a55a79df-703c-46a7-9af8-c9e128d2a3bc.

## Author contributions

DE and CZ conceived the project. All authors conducted the collection. DS wrote the manuscript with support from DE, NA, and CZ. All authors contributed to the article and approved the submitted version.
